# Divining Rods

**Published:** 1876-05

**Authors:** 


### DIVINING RODS.

WITH wonder and awe in the days of our childhood have we stood gazing at a man walking over the ground with measured pace and serious countenance, holding firmly in either hand a prong of a peach-tree twig, uniting in a forked end, which was uppermost. As he passed along in a certain direction the upper end would turn downwards, fairly twisting a prong off in the fingers of some stout unbeliever. We were told that where the twig turned downwards, there was the water. There our well was dug, and there water was found. There was the fact, and off and on we have thought of it ever since, in even doubt whether it was an imposture, a coincidence, or a reality.

